# Periodic open and closed resonators as a biosensor using two computational methods

**DOI:** 10.1038/s41598-024-61987-3

**Published:** 2024-05-24

**Authors:** Zaky A. Zaky, M. Al-Dossari, Ahmed S. Hendy, Wael M. Badawy, Arafa H. Aly

**Affiliations:** 1 TH-PPM Group, Physics Department, Faculty of Science, Beni-Suef University, Beni-Suef, 62521 Egypt; 2 Department of Physics, Faculty of Science, King Khalid University, 62529 Abha, Saudi Arabia; 3 Department of Computational Mathematics and Computer Science, Institute of Natural Sciences and Mathematics, Ural Federal University, 19 Mira St., Yekaterinburg, Russia 620002; 4 Frank Laboratory of Neutron Physics, Joint Institute for Nuclear Research, Dubna, Russian Federation 141980; 5 Radiation Protection and Civil Defense Department, Nuclear Research Center, Egyptian Atomic Energy Authority, Cairo, 13759 Egypt

**Keywords:** Acoustic waves, Gas sensor, Parallel resonators, Phononic crystal, Carbon dioxide concentration, Biomedical engineering, Computational science, Computational methods, Acoustics

## Abstract

The volatile particles and molecules in our dry exhaled breath can reveal enormous information about the health of any person, such as the person’s respiratory and metabolic functioning. Beyond the carbon dioxide level is an indicator of life, it provides important health-related data like people’s metabolic rate. This study considers periodic open and closed resonators for measuring carbon dioxide concentration in dry exhaled breath. Transfer matrix and green methods are used to simulate the interaction between acoustic waves and the proposed sensor. The band gaps using the green method coincide with the transmittance spectra by the transfer matrix. The suggested sensor recorded a sensitivity of $5.3 Hz.{m}^{-1}.s$, a figure of merit of 10,254 ${m}^{-1}.s$, a detection limit of $5\times {10}^{-6} m.{s}^{-1}$, and a quality factor of $3\times {10}^{6}$. Furthermore, the efficiency shows that the proposed design is appropriate as a diagnostic sensor for different diseases such as chronic obstructive pulmonary. Besides, cylindrical-adapted sensors are urgently needed in medicine, industry, and biology because they can simultaneously be used for fluid transport and detection.

## Introduction

Biosensor system detects and quantifies a specific analyte in samples using physicochemical components^1,2^. A biomaterial can be a specific recognition element for analytes, such as enzymes, antibodies, cells, and tissues^3,4^. As a result of the recognition event, the transducer undergoes a physical or chemical change, that yields a detectable optical or electrical signal^5,6^. Many biosensor applications include medical diagnosis, environmental monitoring, and food safety^7–9^. There are several applications for biosensors, including diagnostics, food safety, and environmental defense. Detecting diseases like diabetes, cancer, and infectious diseases has been made possible by biosensors in clinical diagnosis. Food toxins and contaminants, such as pesticides and mycotoxins, have been detected by biosensors in the food industry^10^. As part of environmental monitoring, biosensors have been used to measure pollutants in air, water, and soil^11–13^.

In recent decades, biosensors have been employed to diagnose diseases^14^. Using conventional check-ups or more sophisticated methods like biosensors is necessary to track changes in an individual's health status over time. Biosensors are widely used to monitor chronic diseases like diabetes, heart disease, cancer, etc. Infectious diseases can also be detected with them^15^. Due to their unique optical properties, photonic crystals (PCs) have recently gained significant attention for their use as biosensors^16^. PCs are periodic nanostructures that control light flow by creating a photonic band gap (PBG), which restricts light propagation at specific frequencies. This property enhances the sensitivity and specificity of biosensors.

An enzyme or antibody can be attached to the surface of a PC biosensor to recognize biological elements^16^. When the target molecule binds to the recognition element at the surface, the refractive index (RI) at the surface changes. This change in RI affects the transmission spectrum of the crystal, allowing for the quantification of the target molecule present in a sample^17^. Using PCs as RI sensors is one of the most promising applications of PCs in biosensors. The transmission or reflection of light through PCs can be used to measure RI changes in biological samples, such as changes in the concentration of analytes. Biosensors based on this property have been developed for various biological analytes, including proteins, DNA, and small molecules^18^. In addition, PC biosensors have been demonstrated in several studies to detect biomarkers for cancer^19^, toxins, and pathogens, as well as food analysis and environmental monitoring^16^.

In 1988, Tamura et al.^20^ developed phononic crystals (PnCs) after being inspired by PCs to control how elastic waves propagate in periodic designs. PnCs are manufactured shapes with periodic variations in material properties such as mass, stiffness, tension, etc.^21,22^. This regular change in acoustic properties leads to a phononic band gap (PnBG) analogous to the PBG in PCs^23,24^. Within the PnBG, phonons with specific frequencies aren’t allowed to propagate. Because of the capabilities of PnCs to manipulate acoustic spectra, they are used in various applications, such as acoustic shielding^25^, waveguiding^26^, and biosensors^27^. Therefore, the vast majority of these applications strongly depend on the PnBG. For example, Zaky et al.^27^ investigated a model using regular tubes with different cross-sections for hazardous gas detection. A sensitivity of 2.5495 $Hz {m}^{-1}s$, Q-factor of 4077, and FoM of 9.16 ${m}^{-1}s$ were recorded.

Dry exhaled breath (DEB) primarily comprises oxygen, nitrogen, carbon dioxide, and inert gases^28^. Other small volatile organic compounds (VOC) ratios comprise the rest of the DEB. VOCs in DEB are either formed by cellular metabolic processes or absorbed/inhaled from environmental sources^29^. For example, chronic obstructive pulmonary (COP)^30^, Lung cancer cells^28^, congestive heart failure (CHF)^30^, etc., may produce these VOCs. Due to the low solubility of VOCs in blood, they spread quickly into the exhaled breath^31^.

Detecting carbon dioxide (${CO}_{2}$) concentration in DEB can give non-invasive information about an individual's respiratory and metabolic functioning^31^. Bhagya et al.^30^ proposed a capnographic sensor to sense ${CO}_{2}$ concentration in DEB based on an acoustic virial equation to diagnose COP and CHF diseases. Oort et al.^32^ investigated the vital role of analyzing DEB pneumonia detection. Mazzone et al.^28^ studied the detection of VOC in DEB as a lung cancer biomarker.

The proposed model is unique in the diagnostic field based on detecting ${CO}_{2}$ in DEB. Furthermore, the 1D-PnC with open and closed periodic parallel resonators shows promising results in the diagnostic field. Also, the suggested detector is able to be utilized for monitoring the level of dangerous pollutants flowing through industrial chimneys on a continual basis. This type of detector is easier to fabricate than nanosensors, which need sophisticated and costly manufacturing processes. Besides, cylindrically adapted sensors are urgently needed in medicine, industry, and biology because they can be used simultaneously for fluid transport and detection.

## Materials and mathematical methods

**Figure 1:**
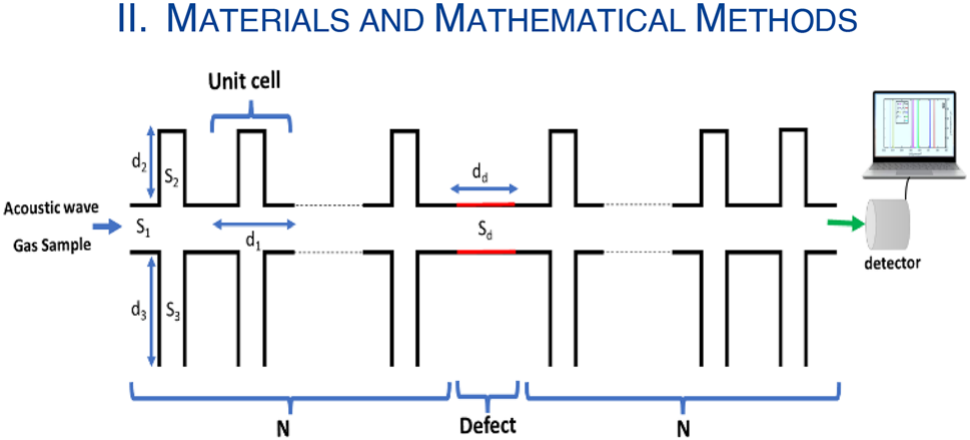
Geometrical representation of the 1D-PnC with open and closed periodic parallel resonators of PVC polymer.

Figure 1 shows a geometrical representation of the 1D-PnC with open and closed periodic parallel resonators of PVC polymer. The proposed design is composed of a duct with a cross-section of ${S}_{1}$, the closed parallel resonators on one side have a cross-section of ${S}_{2}$, and the opened parallel resonators on the other side have a cross-section of ${S}_{3}$. The length of the duct of each cell is ${d}_{1}$, the length of the closed branch is ${d}_{2}$, and the length of the opened branch is ${d}_{3}$. A defect guide with the same cross-section of the main duct and length of ${d}_{d}$ is at the center of the 1D-PnC sensor with open and closed periodic parallel resonators. The main duct, closed branches, and opened branches will be filled with a stationary sample of exhaled breath containing different concentrations of ${CO}_{2}$. These ducts and branches can be made of aluminium and nylon^33^.

This model will neglect the effects of temperature gradients and viscosity by considering the sample is stationary “relatively static” at room temperature^34^. Firstly, all opened resonators will be closed. Then, the DEB sample will fill the model. After that, the resonators will be opened. As a result, the excess gas will leak through the opened branches until the equilibrium state (the outside pressure equals the inside pressure). Currently, the sample is stationary.

### Using the transfer matrix method

As the transfer matrix method (TMM) is easy, fast, and gives the exact dispersion relation of the Green method^35^, the simulations of the response of 1D-PnC with opened and closed periodic parallel resonators of the proposed model to the acoustic waves will be carried out by the TMM^14,36^:

1 $${M}_{i}=\left[\begin{array}{cc}{A}_{i}& {B}_{i}\\ {C}_{i}& {D}_{i}\end{array}\right]\left[\begin{array}{cc}1& 0\\ {y}_{OC}& 1\end{array}\right]\left[\begin{array}{cc}{A}_{i}& {B}_{i}\\ {C}_{i}& {D}_{i}\end{array}\right],$$

2 $${A}_{i}=\mathit{cos}\left(k\frac{{d}_{i}}{2}\right),$$

3 $${B}_{i}=j {Z}_{i} sin\left(k\frac{{d}_{i}}{2}\right),$$

4 $${C}_{i}=\frac{j}{{Z}_{i}}sin\left(k\frac{{d}_{i}}{2}\right),$$

5 $${D}_{i}={A}_{i}.$$

where $c$ is the acoustic speed, $k=\omega /c$, $\rho$ is the density, and the impedance is calculated as the following:

6 $${Z}_{i}=1/{Y}_{i}=\rho c/{{\text{S}}}_{{\text{i}}}.$$

The total acoustic admittance ($${y}_{OC})$$ of the opened ($${y}_{O})$$ and closed ($${y}_{C})$$ resonators to the acoustic wave are calculated as:

7 $${y}_{O}=-j \frac{1}{{Z}_{3}} cot\left(k{d}_{3}\right),$$

8 $${y}_{C}=j \frac{1}{{Z}_{2}} tan\left(k{d}_{2}\right),$$

9 $${y}_{OC}={y}_{O}+{y}_{C}.$$

For the defect cell:

10 $${M}_{D}=\left[\begin{array}{cc}{A}_{d}& {B}_{d}\\ {C}_{d}& {D}_{d}\end{array}\right],$$

11 $${A}_{d}=\mathit{cos}\left(k\frac{{d}_{d}}{2}\right),$$

12 $${B}_{d}=j {Z}_{d} sin\left(k\frac{{d}_{d}}{2}\right),$$

13 $${C}_{d}=\frac{j}{{Z}_{d}}sin\left(k\frac{{d}_{d}}{2}\right),$$

14 $${D}_{d}={A}_{d},$$

where ${Z}_{d}=\rho c/{{\text{S}}}_{{\text{d}}}$. The transmittance (T) of the 1D-PnC with open and closed periodic parallel resonators is computed as the following:

15 $$t=\frac{2{Y}_{1}}{\left({A}_{11}+{A}_{12}{Y}_{1}\right){Y}_{1}+\left({A}_{21}+{A}_{22}{Y}_{1}\right)},$$

16 $$T\left(\%\right)=100\times {\left|t\right|}^{2}.$$

### Using Green method

Using Bloch’s theorem, the band structure of 1D-PnC with open and closed periodic parallel resonators was investigated and matched the TMM results^35^. In the case of 1D-PnC with open and closed periodic parallel resonators, the function of Green’s surface (GS) of an elementary cell ${g}_{R}^{-1}\left(\mathrm{0,0}\right)$ is:

17 $${g}_{R}^{-1}\left(\mathrm{0,0}\right)={g}_{c}^{-1}\left(\mathrm{0,0}\right)+{g}_{o}^{-1}\left(\mathrm{0,0}\right),$$

where ${g}_{c}^{-1}\left(\mathrm{0,0}\right)$, and ${g}_{o}^{-1}\left(\mathrm{0,0}\right)$ are the function of GS of one cell of closed and opened resonator as the following:

18 $${g}_{c}^{-1}\left(\mathrm{0,0}\right)= - j {y}_{2}\mathit{tan}\left(k{d}_{2}\right),$$

19 $${g}_{o}^{-1}\left(\mathrm{0,0}\right)= - j {y}_{2}\mathit{Cotan}\left(k{d}_{2}\right).$$

The dispersion for the infinite waveguide is:

20 $$\mathit{cos}\left(Kd\right)= \mathit{cos}\left(k{d}_{1}\right)- \frac{1}{2}\frac{{z}_{1}}{\omega }\mathit{sin}\left(k{d}_{1}\right){g}_{R}^{-1}\left(\mathrm{0,0}\right),$$

21 $$\mathit{cos}\left(Kd\right)= \mathit{cos}\left(k{d}_{1}\right)+ \frac{j}{2}{Z}_{1}{Y}_{OC}\mathit{sin}\left(k{d}_{1}\right),$$

where $K$ and $k$ are the Bloch and wave vectors, respectively. All results are performed using MATLAB software on a laptop with the configuration: Intel(R) Core (TM) i5 CPU, 16:00 G RAM, and 2:67 GHz, with 64 bits operating system. The elapsed computation time for every run is about 8 s.

Laly et al.^37^ studied homogeneous and heterogeneous acoustic absorbing metamaterials using TMM and the finite element method (FEM). In their studies, the results using TMM and FEM were coinciding. Almeida et al.^38^ designed intake mufflers with different cross-sections. Almeida et al. compared the numerical results using TMM and experimental results. The numerical results recorded high matches.

The effective acoustic speed of the mixture of gases (${c}_{mix}$) of different concentrations ($\alpha$) can be calculated as the following^39^:

22 $${c}_{mix}=\frac{\sum {\alpha }_{i}{\rho }_{i}{C}_{i}}{\sum {\alpha }_{i}{\rho }_{i}},$$

where $\rho$ is the mass density and $c$ is the acoustic speed of each gas sample. The effective mass density of the mixture of gases (${\rho }_{mix}$) can be calculated as the following^40^:

23 $${\rho }_{mix}=\sum {\alpha }_{i}{\rho }_{i}.$$

## Results and discussions

At first, the geometrical dimensions of the proposed sensors' main duct, the closed branches, and the opened branches will be N = 10, ${d}_{1}$ = 10 cm, ${d}_{2}$ = ${d}_{3}$  = 6 cm, ${d}_{d}$ = 33 cm, ${S}_{d}$ = ${S}_{1}$  = 1 ${cm}^{2}$, ${S}_{2}$= ${S}_{3}$=0.9 ${cm}^{2}$. For normal humans, the DEB is composed of a mixture of ${N}_{2}$ (78%), ${O}_{2}$ (16%), ${CO}_{2}$ (5%), and Ar (1%). Table 1 contains $c$ and $\rho$ of pure gas samples to calculate the effective $c$ and $\rho$ of the DEB samples according to Eqs. (22) and (23).

**Table 1:** Acoustic speed and density of pure gases^23,41^.

Gas sample	Density (kg/${m}^{3}$)	Acoustic speed (m/s)
${CO}_{2}$	1.8393	267
Ar	1.661	319
${O}_{2}$	1.314	326
Air	1.2047	343
${N}_{2}$	1.165	349

**Figure 2:**
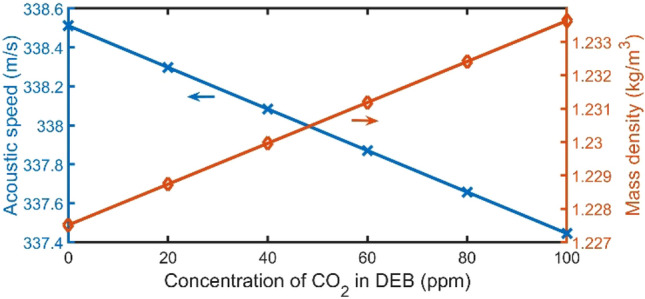
The acoustical velocity of the mixture and its mass density versus the ratio of ${CO}_{2}$.

**Figure 3:**
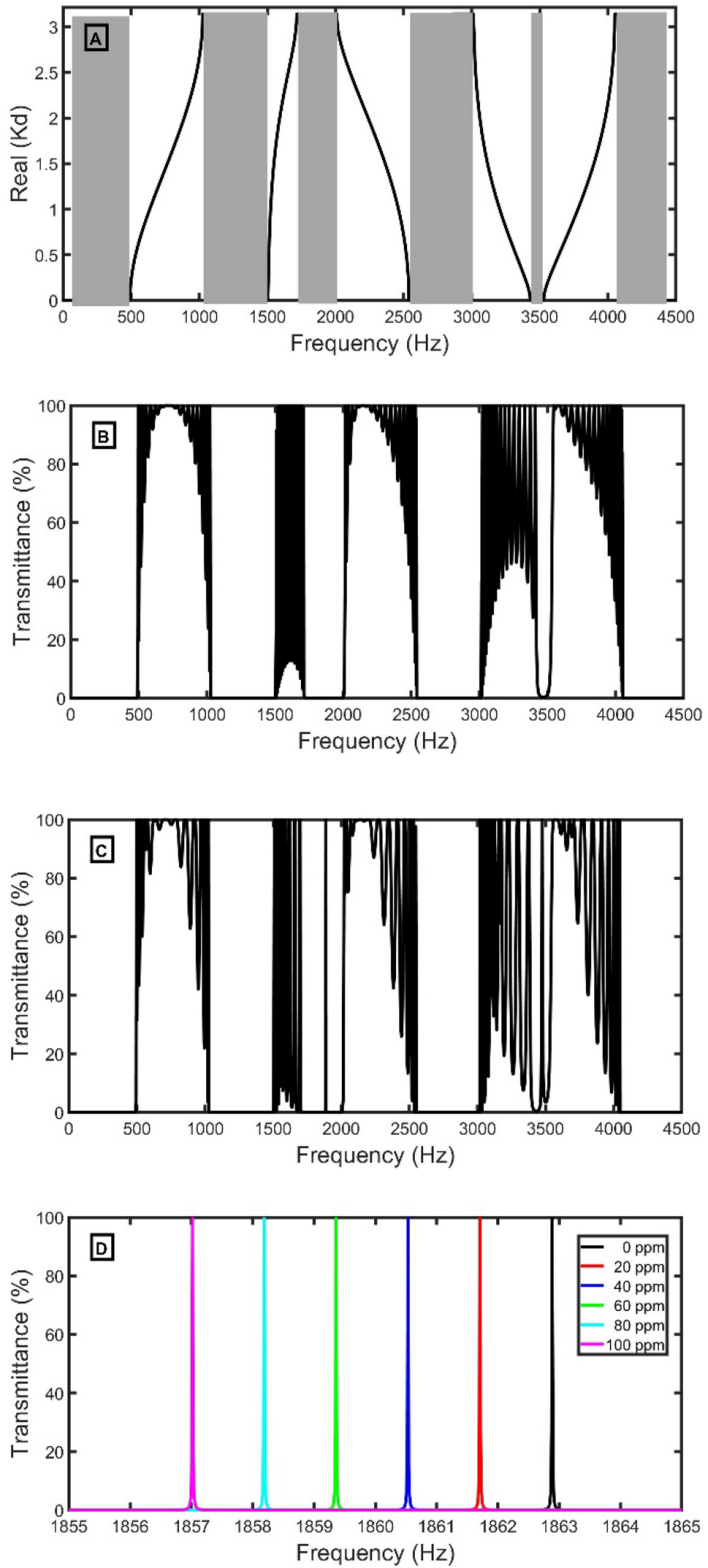
(**A**) Band structure of a unit cell using the Green method, (**B**) Transmittance of perfect open and closed periodic parallel resonators using a sample of air, (**C**) the transmittance of the defected 1D-PnC with open and closed periodic parallel resonators using a sample of air, and (**D**) Transmittance of defected open and closed periodic parallel resonators using a sample of exhaled breath with different concentrations of ${CO}_{2}$.

As clear in Fig. 2, the acoustic speed and density of the typical DEB are 338.51 m/s and 1.2275 kg/${m}^{3}$, respectively. By increasing the concentration of ${CO}_{2}$ higher than the usual ratio in DEB, the $c$ of the mixture regularly decreases, and the $\rho$ of the mixture regularly increases. Figure 3A shows the band structure of a unit cell of the proposed 1D-PnC with open and closed periodic parallel resonators using an air sample. A DEB sample will be pushed inside the structure. The excess in the DEB sample inside the structure will leak through the opened tubes (stationary state). The DEB sample will go through a dryer to dry water and absorb other organic components from the sample, as clear in the graphical abstract.

The band structure using Bloch’s theorem consists of PnBGs that coincide with the PnBGs in the transmittance spectrum using TMM in Fig. 3B. Due to the step change in the acoustic impedance inside the sensor before and at the resonators, multiple Bragg scattering forms the PnBGs. A defect peak penetrates the third PnBG at 1887.59 Hz by including a horizontal defect cell because of the acoustic wave's localization, as evident in Fig. 3C. Figure 3D clarifies that the defect peak has red-shifted from 1862.89 to 1861.71 Hz, 1860.53 Hz, 1859.36 Hz, 1858.19 Hz, and 1857.02 Hz due to the variation of the ${CO}_{2}$ concentration from 0 to 100 ppm in the DEB above the normal ratio. According to Table 1 and the standing wave relation, increasing the ${CO}_{2}$ concentration decreases the acoustic speed, and the resonant frequency decreases. As a result, the resonant peaks are red-shifted to lower frequencies.

24 $$2d=\frac{n c}{{f}_{R}}.$$

The main goal of the following calculations is to select the relatively best conditions. Therefore, the most important parameters to evaluate the quality of sensors are the transmittance of resonance (T), sensitivity (S), the bandwidth of the resonant peak (FWHM), the figure of merit (FoM), quality factor (Q), and detection limit (LoD), as the following^42^:

25 $$S =\frac{\Delta {f}_{R}}{\Delta c},$$

26 $$FoM=\frac{S}{FWHM},$$

27 $$\begin{array}{c}Q=\frac{{f}_{R}}{FWHM}\end{array},$$

28 $$LoD=\frac{{f}_{R}}{20 S Q},$$

where ${f}_{R}$ is the peak frequency. The signal-to-noise ratio (SNR) and resolution (RS) will be calculated as follows^43^:

$$SNR=\frac{\Delta {f}_{R}}{FWHM},$$

$$RS=\frac{2 FWHM}{3{(SNR)}^{0.25}}.$$

The resolution of any sensor device reflects the smallest change in the measurable peak that can be detected accurately.

**Figure 4:**
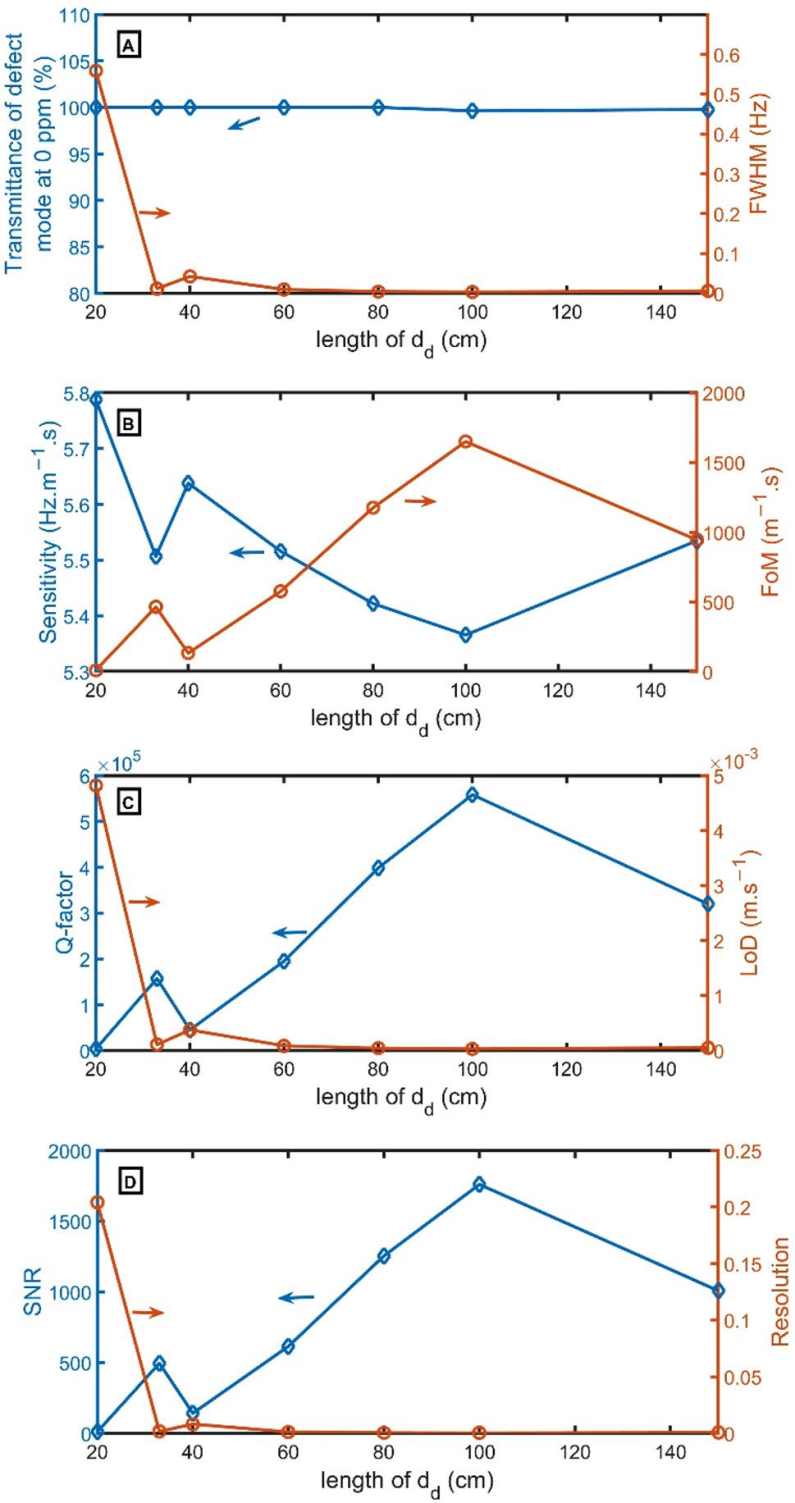
(**A**) Transmittance at 0 ppm (left axis) and FWHM (right axis), (**B**) Sensitivity (left axis) and FoM (right axis), (**C**) Q-factor (left axis) and LoD (right axis), and (**D**) SNR (left axis) and RS (right axis) versus the length of ${d}_{d}$.

In Fig. 4A–D, T, S, FWHM, Q, FoM, LoD, SNR and RS are calculated versus ${d}_{d}$. Transmittance and FWHM of defect mode at a concentration of ${CO}_{2}$ of 0 ppm concentration indicates the intensity and bandwidth of peaks, as clear in Fig. 4A. As ${d}_{d}$ varies from 20 to 150 cm, the transmittance has a value very close to 100%, and FWHM changes from 0.558 to 0.006 Hz. The narrower FWHM of 0.003 Hz is achieved at ${d}_{d}$ = 100 cm. The sensitivity is studied based on the change of ${f}_{R}$ with the change $c$ of ${CO}_{2}$ concentration from 0 to 100 ppm, as shown in Fig. 4B. At the thickness ${d}_{d}$ = 100 cm, the resonant peak has a high transmittance of 99.6% and the lowest FWHM of 0.003 Hz. Besides, the FoM, Q-factor, and SNR have the highest value, but LoD and RS have the lowest value. So, ${d}_{d}$ = 100 cm is the selected thickness.

**Figure 5:**
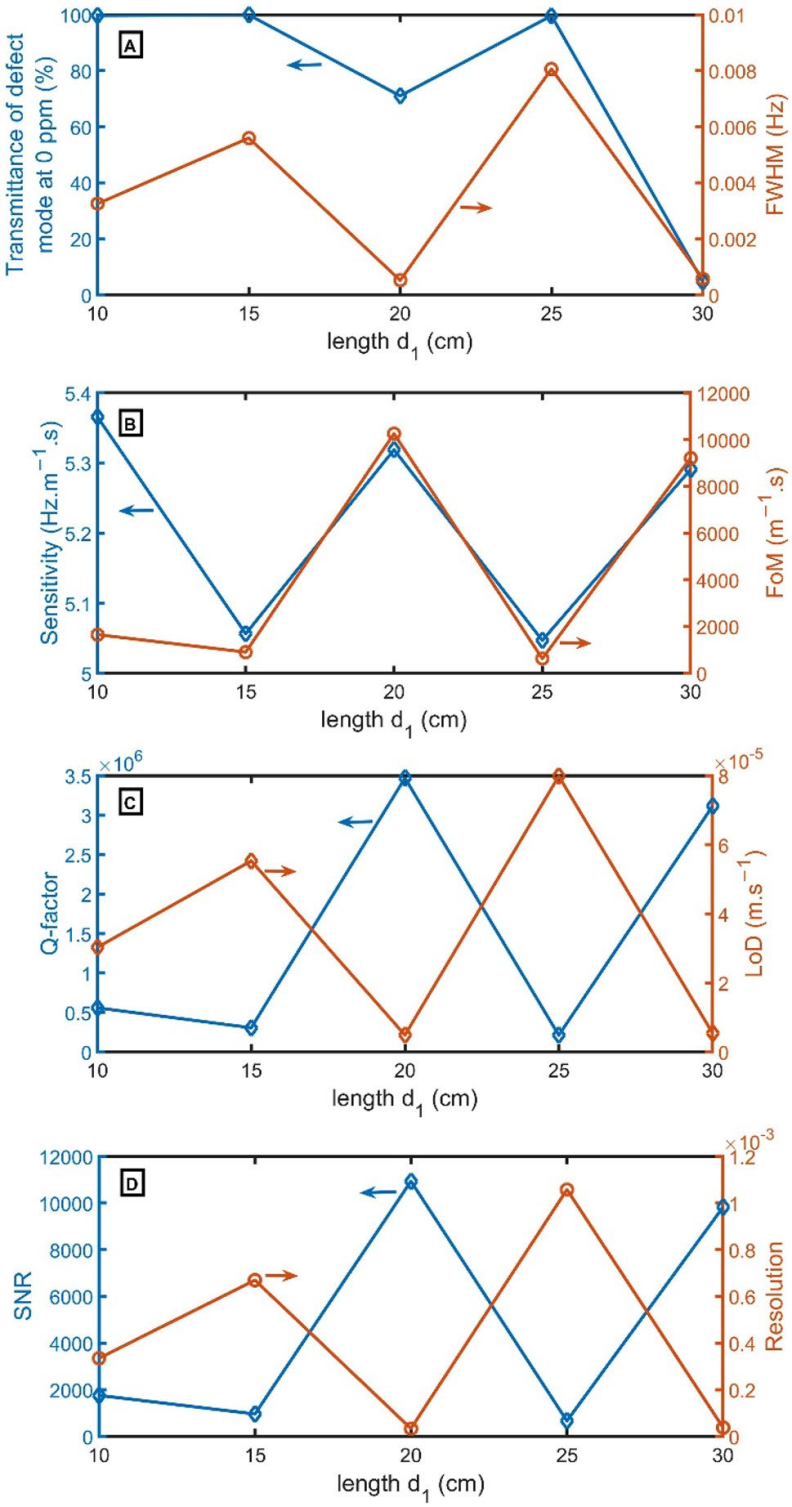
(**A**) Transmittance at 0 ppm (left axis) and FWHM (right axis), (**B**) Sensitivity (left axis) and FoM (right axis), (**C**) Q-factor (left axis) and LoD (right axis), and (**D**) SNR (left axis) and RS (right axis) versus the length of ${d}_{1}$.

In Fig. 5A, at ${d}_{1}$ of 10 cm, 15 cm, and 25 cm, the transmittance becomes 100%. But at ${d}_{1}$ of 20 cm and 30 cm, the transmittance becomes 71% and 5%, respectively. Moreover, the FWHM of the resonant peak is also given on the right axis of Fig. 5A. At ${d}_{1}$ of 20 cm, the resonant peak has the narrowest FWHM of 0.0005 Hz. As explicit in Fig. 5B–D, the FoM, Q-factor, and SNR have the highest performance of 10,254 ${m}^{-1}.s$, 3,470,288, and 10,930, respectively, at ${d}_{1}$ of 20 cm, but LoD and RS have the lowest value of 5 $\times {10}^{-3} m.{s}^{-1}$, and 3 $\times {10}^{-5}$. For sensitivity, the highest values are achieved at ${d}_{1}$ of 10 cm and then 20 cm. As a result, ${d}_{1}$ of 20 cm will be selected.

**Figure 6:**
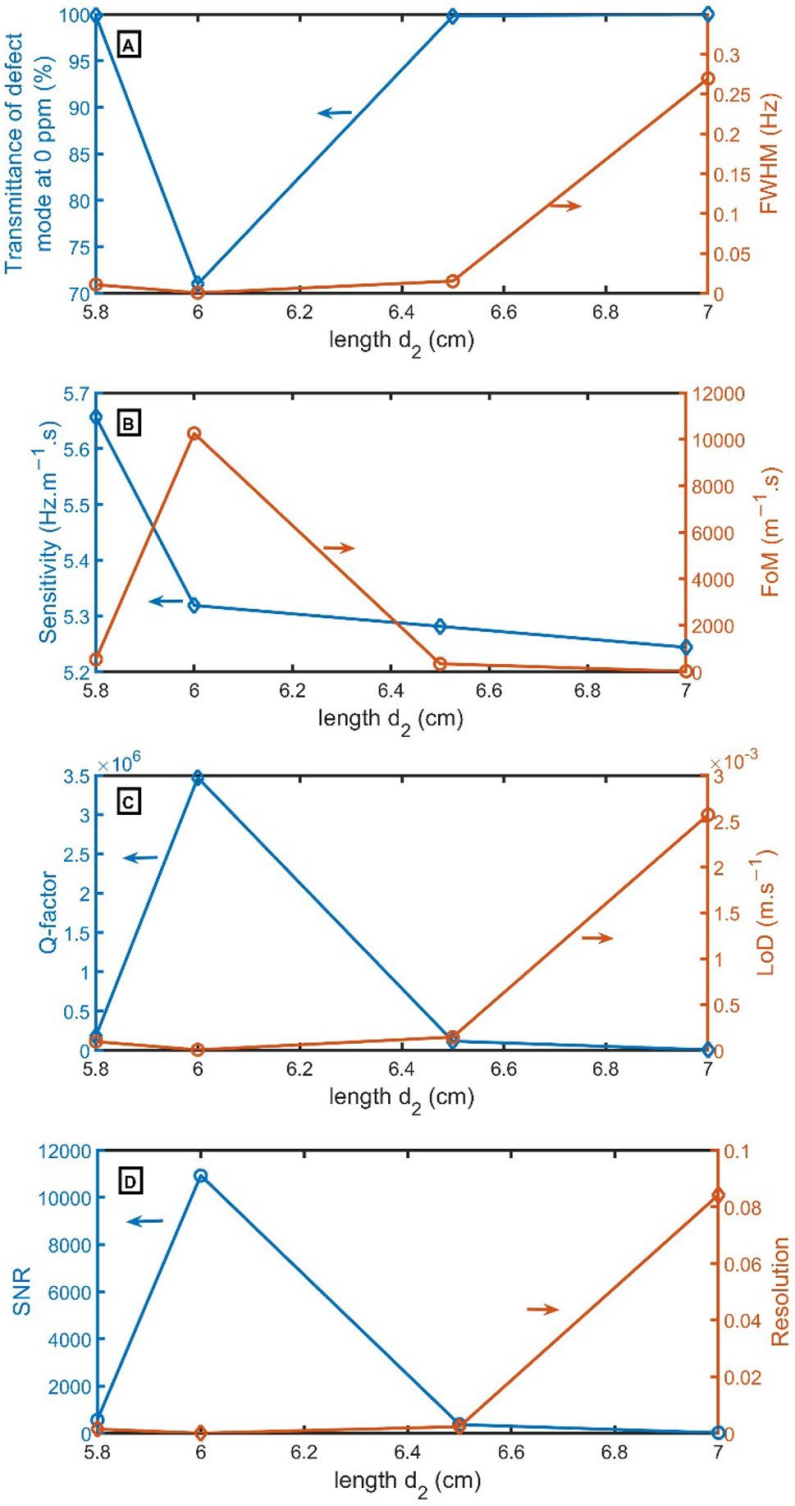
(**A**) Transmittance at 0 ppm (left axis) and FWHM (right axis), (**B**) Sensitivity (left axis) and FoM (right axis), (**C**) Q-factor (left axis) and LoD (right axis), and (**D**) SNR (left axis) and RS (right axis) versus the length of ${d}_{2}$.

From Fig. 6A, the transmittance has maximum values at ${d}_{2}$ of 5.8 cm, 6.5 cm, and 7.0 cm. But transmittance of 71% is recorded at ${d}_{2}$ of 6.0 cm. At thicknesses of ${d}_{2}$ higher or lower than these selected values, the resonant mode disappears. At ${d}_{2}$ of 6.0 cm, the resonant peak has the narrowest FWHM of 0.0005 Hz. As explicit in Fig. 6B–D, the FoM, Q-factor, and SNR have the highest performance of 10,254 ${m}^{-1}.s$, 3,470,288, and 10,931, respectively, at ${d}_{2}$ of 6.0 cm, but LoD and RS have the lowest value of $5\times {10}^{-6}$ $m.{s}^{-1}$, and $2\times {10}^{-3}$. For sensitivity, the highest values are achieved at ${d}_{2}$ of 5.8 cm and then 6.0 cm. As a result, ${d}_{2}$ of 6.0 cm will be selected.

**Figure 7:**
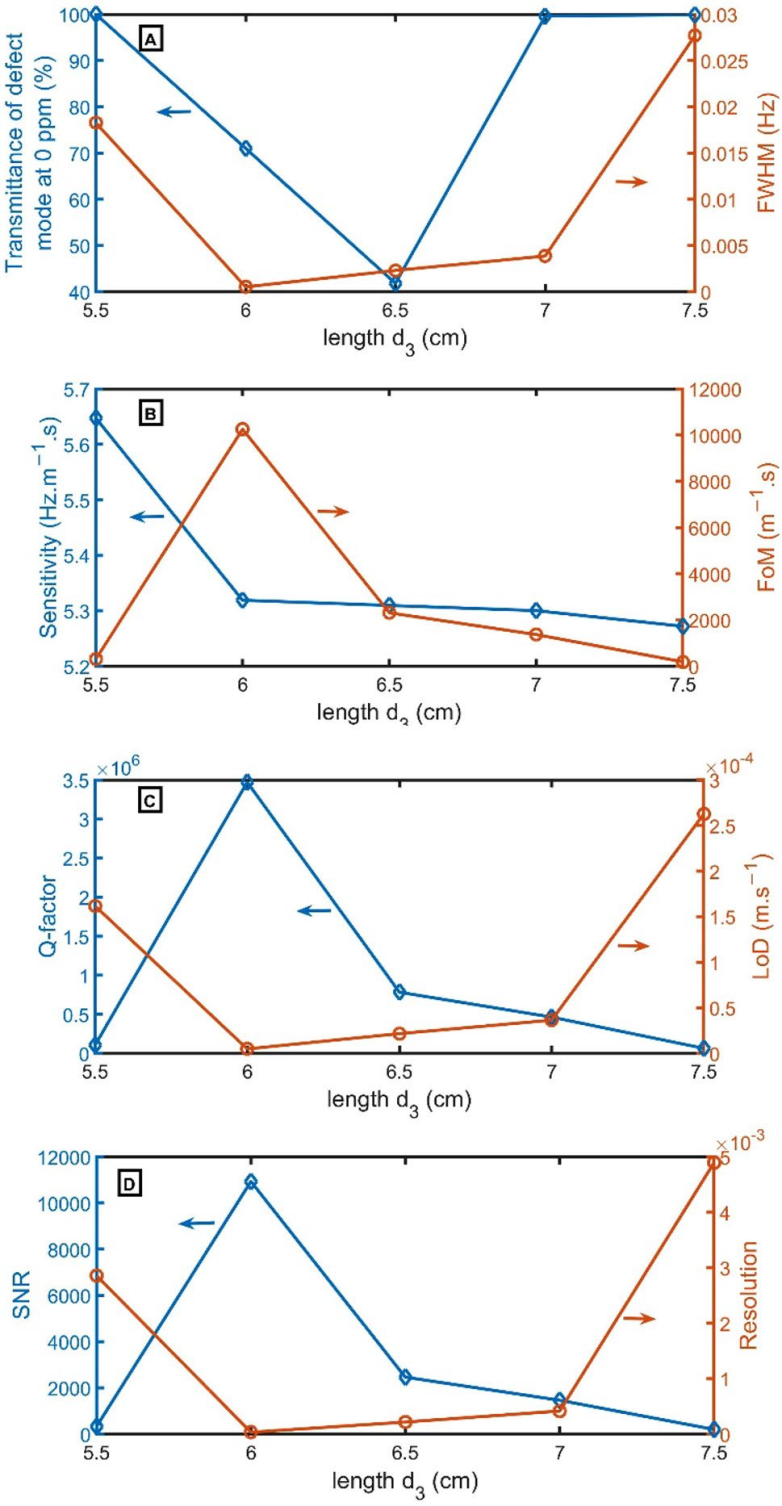
(**A**) Transmittance at 0 ppm (left axis) and FWHM (right axis), (**B**) Sensitivity (left axis) and FoM (right axis), (**C**) Q-factor (left axis) and LoD (right axis), and (**D**) SNR (left axis) and RS (right axis) versus the length of ${d}_{3}$.

Transmittance, FWHM, Q, S, FoM, LoD, SNR, and RS are studied versus the length of ${d}_{3}$, as clear in Fig. 7A–D. As the thickness of ${d}_{3}$ varies from 5.5 to 6.5 cm, the transmittance decreases from 100 to 42%. By increasing ${d}_{3}$ from 6.5 to 7 cm and 7.5 cm, the transmittance increases to 100%. The FWHM decreases from 0.0182 to 0.00052 Hz by changing ${d}_{3}$ from 5.5 to 6.0 cm. But the FWHM increases from 0.00052 to 0.02771 Hz by changing ${d}_{3}$ from 6.0 to 7.5 cm, as apparent in Fig. 7A. Increasing the ${d}_{3}$ from 5.5 to 6.0 cm decreases sensitivity from 5.6 to 5.3 $Hz.{m}^{-1}.s$. Then, it slightly changes with increasing ${d}_{3}$, as clear in Fig. 7B. On the other axis, FoM changes from 309 ${m}^{-1}.s$ to 10,254 ${m}^{-1}.s$, 2310 ${m}^{-1}.s$, 1372 ${m}^{-1}.s$, and 190 ${m}^{-1}.s$, by increasing ${d}_{3}$ from 5.5 cm to 6.0 cm, 6.5 cm, 7.0 cm, and 7.5 cm, respectively. As clear in Fig. 7C, the Q-factor changes from 104,563 to 3,470,288, 782,033, 464,169, and 64,447 by increasing ${d}_{3}$ from 5.5 cm to 6.0 cm, 6.5 cm, 7.0 cm, and 7.5 cm, respectively. On the other axis, the LoD decreases from $1.6\times {10}^{-5}$ to 4.8 $\times {10}^{-6}$ $m.{s}^{-1}$ by changing ${d}_{3}$ from 5.5 to 6.0 cm. But the LoD increases from 4.8 $\times {10}^{-6}$ to 2.6 $\times {10}^{-4} m.{s}^{-1}$ by changing ${d}_{3}$ from 6.0 to 7.5 cm, as apparent in Fig. 7A. At the thickness ${d}_{3}$ = 6.0 cm, the resonant peak has a slightly high transmittance of 71% and the lowest FWHM of 0.0005 Hz. Besides FoM, sensitivity, Q-factor, and SNR have the highest value, but LoD and RS have the lowest value, as clear in Fig. 7B–D. So, ${d}_{3}$ = 6.0 cm is the selected thickness.

**Figure 8:**
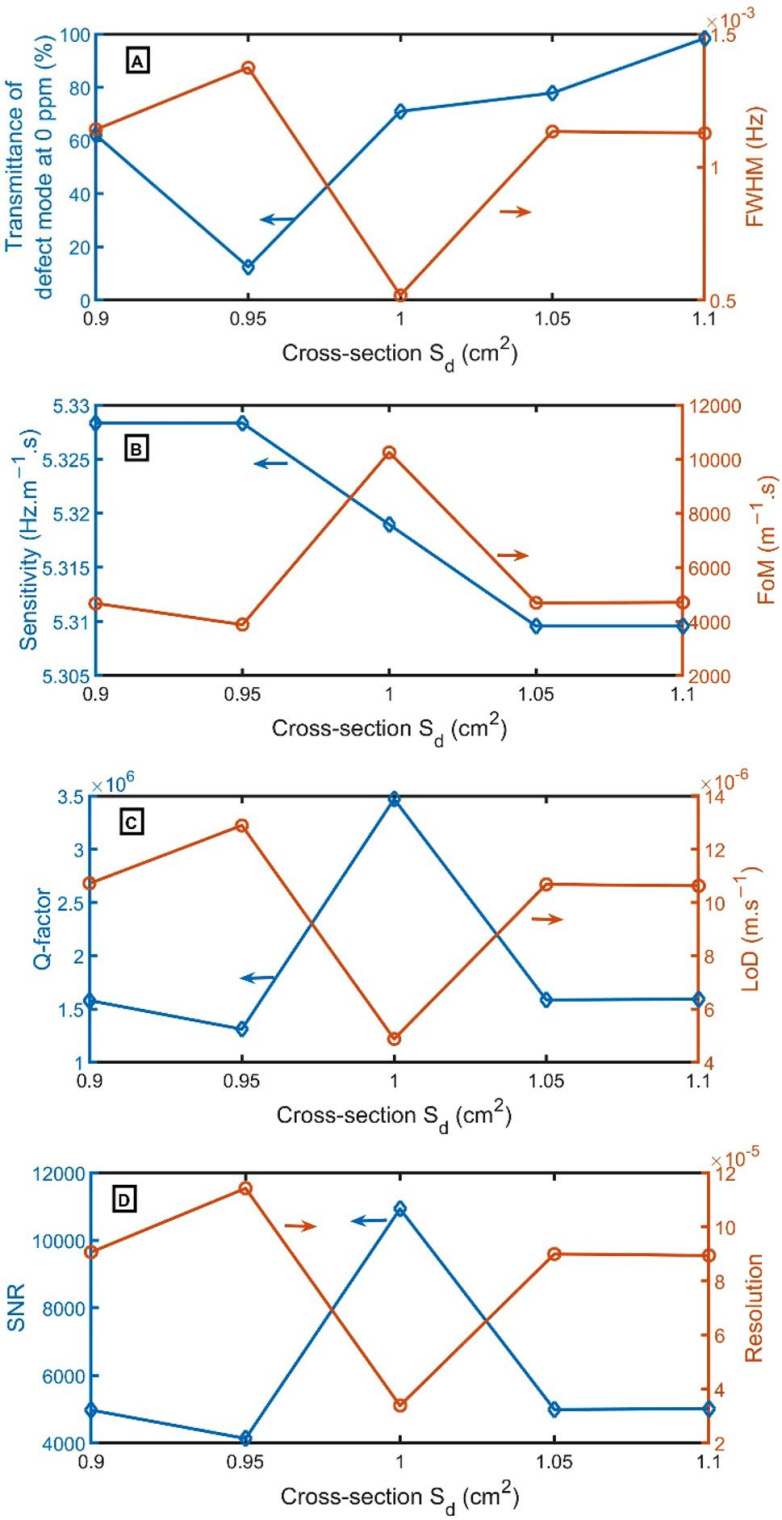
(**A**) Transmittance at 0 ppm (left axis) and FWHM (right axis), (**B**) Sensitivity (left axis) and FoM (right axis), (**C**) Q-factor (left axis) and LoD (right axis), and (**D**) SNR (left axis) and RS (right axis) versus ${S}_{d}$.

According to Fig. 8A, by changing ${S}_{d}$ from 0.90 ${cm}^{2}$ to 0.95 ${cm}^{2}$, 1.00 ${cm}^{2}$, 1.05 ${cm}^{2}$, and 1.10 ${cm}^{2}$, the transmittance of resonant frequencies alters from 62 to 13%, 71%, 78%, and 98%, respectively. At the cross-section of ${S}_{d}$ of 1.00 ${cm}^{2}$, the resonant peak has the narrowest FWHM of 0.0005 Hz. As clear in Fig. 8B–D, the FoM, Q-factor, and SNR have the highest performance of 10,254 ${m}^{-1}s$, 3,470,288, and 10,931, respectively, at ${S}_{d}$ of 1.00 ${cm}^{2}$, but LoD and RS have the lowest value of 5 $\times {10}^{-6}$ $m.{s}^{-1}$, and 3.4 $\times {10}^{-5}$. For sensitivity, by altering ${S}_{1}$ from 0.90 to 0.95 ${cm}^{2}$, the sensitivity has not changed. As a result, ${S}_{d}$ of 1.00 ${cm}^{2}$ will be selected.

**Figure 9:**
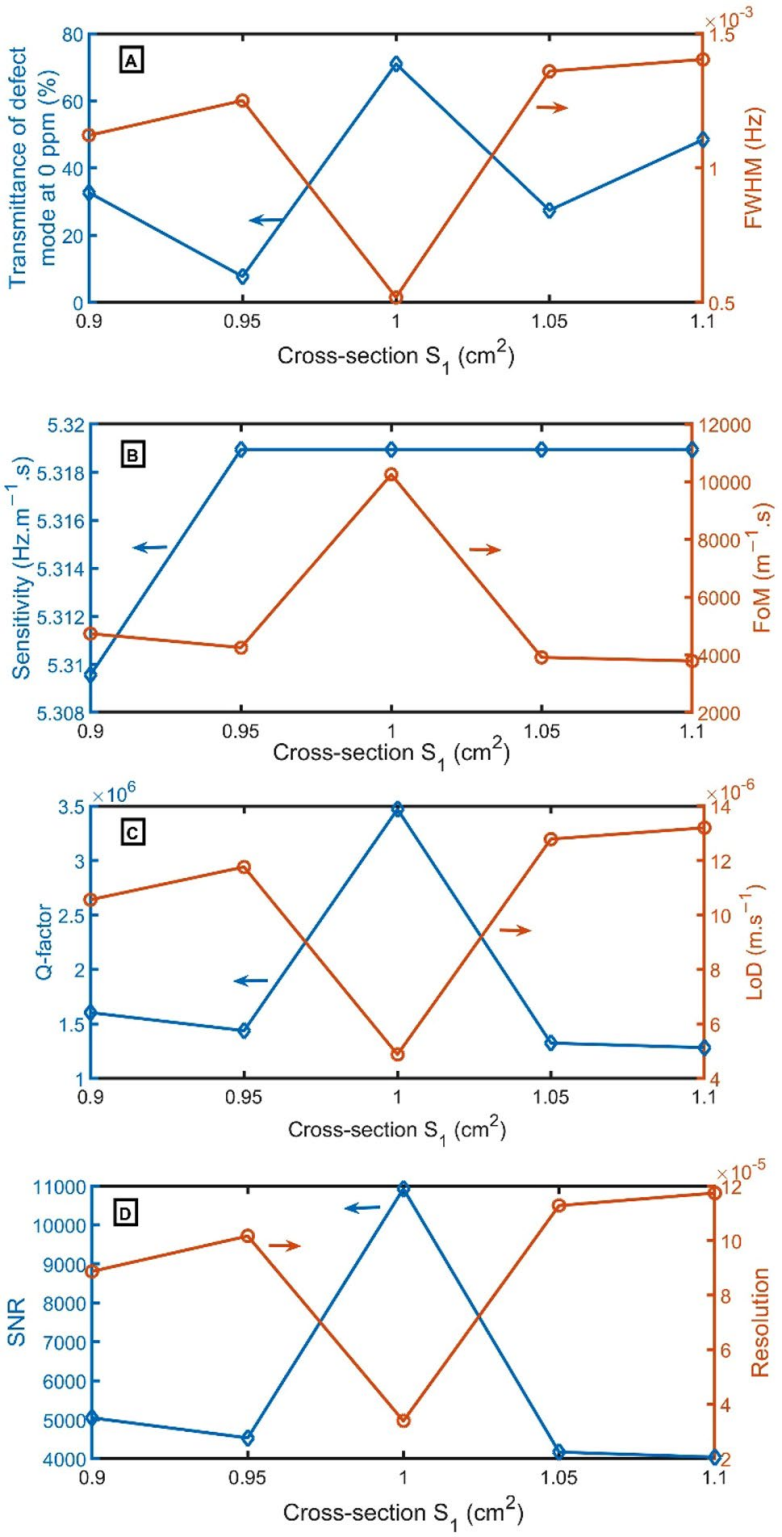
(**A**) Transmittance at 0 ppm (left axis) and FWHM (right axis), (**B**) Sensitivity (left axis) and FoM (right axis), (**C**) Q-factor (left axis) and LoD (right axis), and (**D**) SNR (left axis) and RS (right axis) versus ${S}_{1}$.

In Fig. 9A, by altering the cross-section of ${S}_{1}$ from 0.90 ${cm}^{2}$ to 0.95 ${cm}^{2}$, 1.00 ${cm}^{2}$, 1.05 ${cm}^{2}$, and 1.10 ${cm}^{2}$, the transmittance of resonant frequencies alters from 33 to 8%, 71%, 27%, and 48%, respectively. At the cross-section of ${S}_{1}$ of 1.00 ${cm}^{2}$, the resonant peak has the narrowest FWHM of 0.0005 Hz. As clear in Fig. 9B–D, the FoM, Q-factor, and SNR have the highest performance of 10,254 ${m}^{-1}s$, 3,470,288, and 10,931, respectively, at ${S}_{1}$ of 1.00 ${cm}^{2}$, but LoD and RS have the lowest value of 5 $\times {10}^{-6}$ $m.{s}^{-1}$ and 3 $\times {10}^{-5}$. For sensitivity, by altering ${S}_{1}$ from 0.90 to 0.95 ${cm}^{2}$, S slightly increased from 5.31 $Hz.{m}^{-1}.s$ to 5.32 $Hz.{m}^{-1}.s$. Then, the sensitivity does not change. As a result, ${S}_{1}$ of 1.00 ${cm}^{2}$ will be selected.

**Figure 10:**
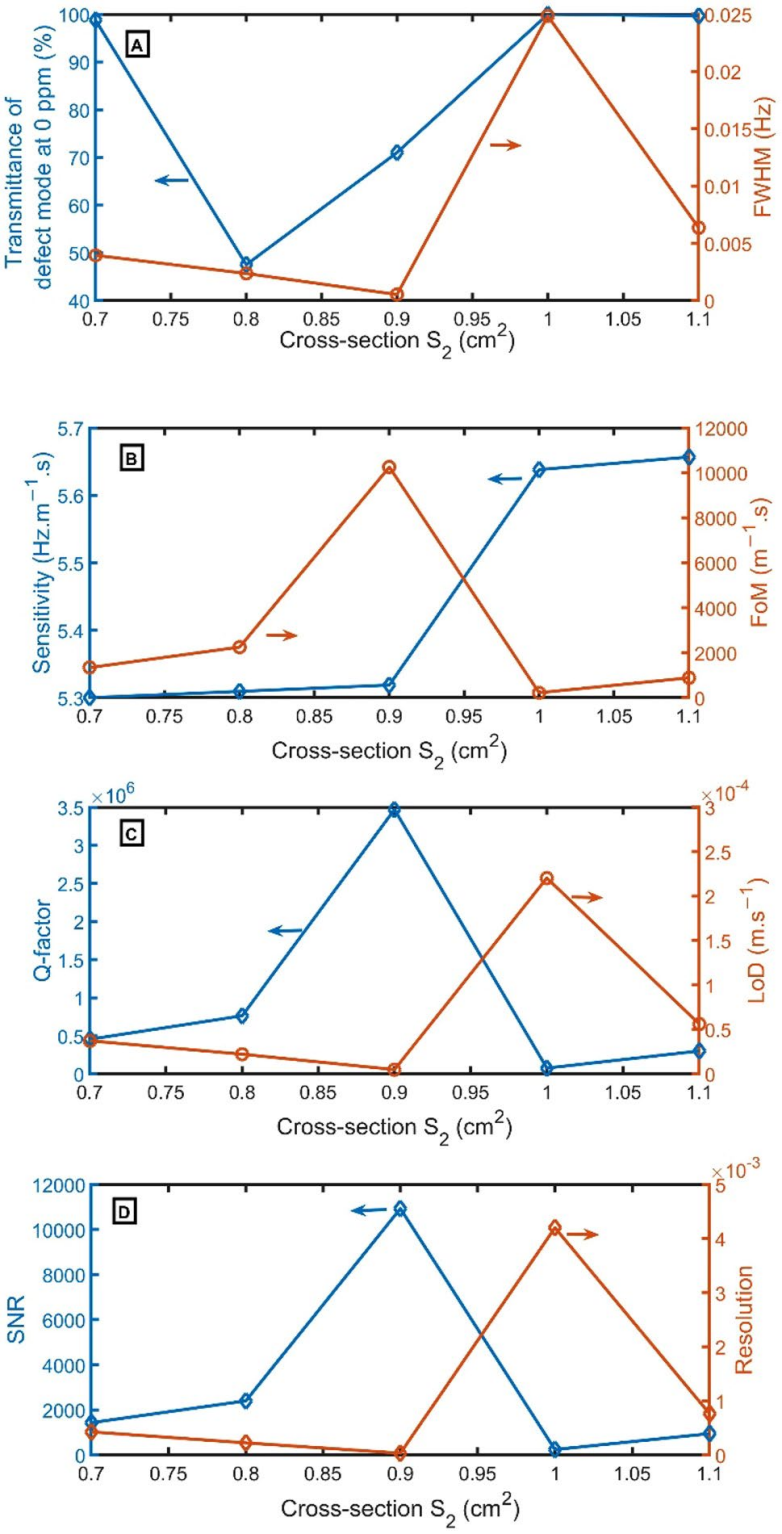
(**A**) Transmittance at 0 ppm (left axis) and FWHM (right axis), (**B**) Sensitivity (left axis) and FoM (right axis), (**C**) Q-factor (left axis) and LoD (right axis), and (**D**) SNR (left axis) and RS (right axis) versus ${S}_{2}$.

In Fig. 10A, at ${S}_{2}$ of 0.7 ${cm}^{2}$, 1.0 ${cm}^{2}$, and 1.1 ${cm}^{2}$, the transmittance becomes 100%. But at ${S}_{2}$ of 0.8 ${cm}^{2}$ and 0.9 ${cm}^{2}$, the transmittance becomes 48% and 71%, respectively. Moreover, the FWHM of the resonant peak is also given on the right axis of Fig. 10A. At ${S}_{2}$ of 0.9 ${cm}^{2}$, the resonant peak has the narrowest FWHM of 0.0005 Hz. As clear in Fig. 10B–D, the FoM, Q-factor, and SNR have the highest performance of 10,254 ${m}^{-1}s$, 3,470,288, and 10,931, respectively, at ${S}_{2}$ of 0.9 ${cm}^{2}$, but LoD and RS have the lowest value of 5 $\times {10}^{-6}$ $m.{s}^{-1}$, and 3 $\times {10}^{-5}$. For sensitivity, the highest values are achieved at ${S}_{2}$ of 1.1 ${cm}^{2}$. As a result, ${S}_{2}$ of 0.9 ${cm}^{2}$ will be selected.

**Figure 11:**
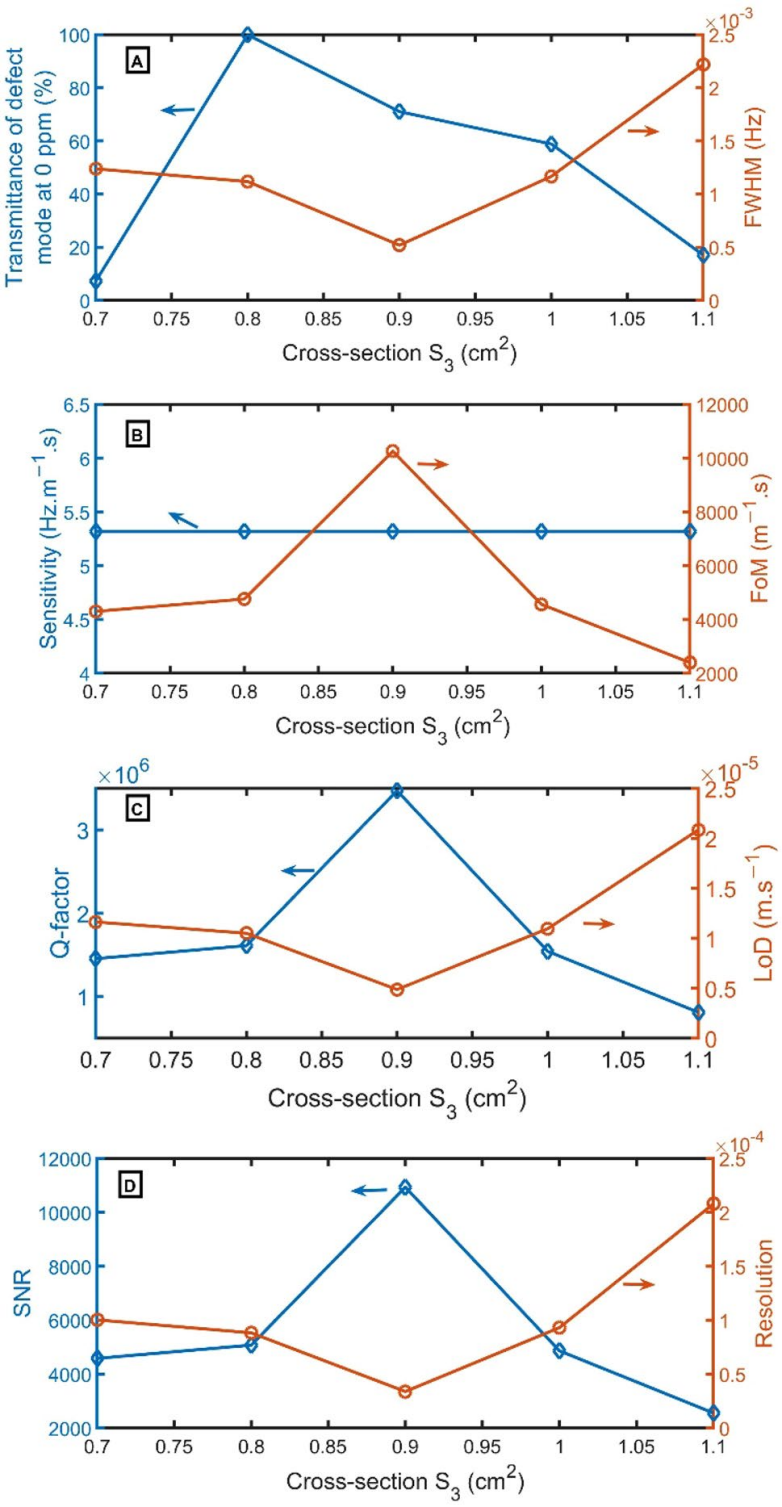
(**A**) Transmittance at 0 ppm (left axis) and FWHM (right axis), (**B**) Sensitivity (left axis) and FoM (right axis), and (**C**) Q-factor (left axis) and LoD (right axis), and (**D**) SNR (left axis) and RS (right axis) versus ${S}_{3}$.

As the cross-section of ${S}_{3}$ varies from 0.7 to 0.8 ${cm}^{2}$, the transmittance increases from 7 to 100%. Then, the transmittance decreases to 71%, 59%, and 17% by increasing ${S}_{3}$ to 0.9 ${cm}^{2}$, 1.0 ${cm}^{2}$, and 1.1 ${cm}^{2}$. The FWHM reduces from 0.00123 to 0.00052 Hz by changing ${S}_{3}$ from 0.7 ${cm}^{2}$ to 0.9 ${cm}^{2}$. But the FWHM grew from 0.00052 to 0.00222 Hz by changing ${S}_{3}$ from 0.9 to 1.1 ${cm}^{2}$, as apparent in Fig. 11A. In Fig. 11B, sensitivity did not change with increasing the ${S}_{3}$ from 0.7 to 1.1 ${cm}^{2}$.

As for FoM increases from 4300 to 10,254 ${m}^{-1}.s$ by changing ${S}_{3}$ from 0.7 to 0.9 ${cm}^{2}$, But it decreases from 10,254 to 2398 ${m}^{-1}.s$ by changing ${S}_{3}$ from 0.9 to 1.1 ${cm}^{2}$. In Fig. 11C, the Q-factor increases from 1,456,134 to 3,470,288 by changing ${S}_{3}$ from 0.7 to 0.9 ${cm}^{2}$, But it decreases from 3,470,288 to 811,123 by changing ${S}_{3}$ from 0.9 to 1.1 ${cm}^{2}$. As for LoD decreases from 1.16 $\times {10}^{-5}$to 4.88 $\times {10}^{-6}$ $m. {s}^{-1}$ by changing ${S}_{3}$ from 0.7 to 0.9 ${cm}^{2}$, But it increases from 4.88 $\times {10}^{-6}$ to 2.08 $\times {10}^{-5}$ $m.{s}^{-1}$ by changing ${S}_{3}$ from 0.9 to 1.1 ${cm}^{2}$. As for SNR, it increases from 4584 to 10,931 by changing ${S}_{3}$ from 0.7 to 0.9 ${cm}^{2}$, But it decreases from 10,931 to 2557 by changing ${S}_{3}$ from 0.9 to 1.1 ${cm}^{2}$. On the other axis, the RS records the lowest value of 3 $\times {10}^{-5}$ at ${S}_{3}$ of 0.9 ${cm}^{2}$, as clear in Fig. 11D.

Therefore, at the cross-section ${S}_{3}$ = 0.9 ${cm}^{2}$, the resonant peak has a slightly high transmittance of 71% and the lowest FWHM of 0.0005 Hz. Besides, the Q-factor and FoM have the highest value, and LoD has the lowest value. So, ${S}_{3}$ = 0.9 ${cm}^{2}$ is the selected cross-section of ${S}_{3}$.

**Figure 12:**
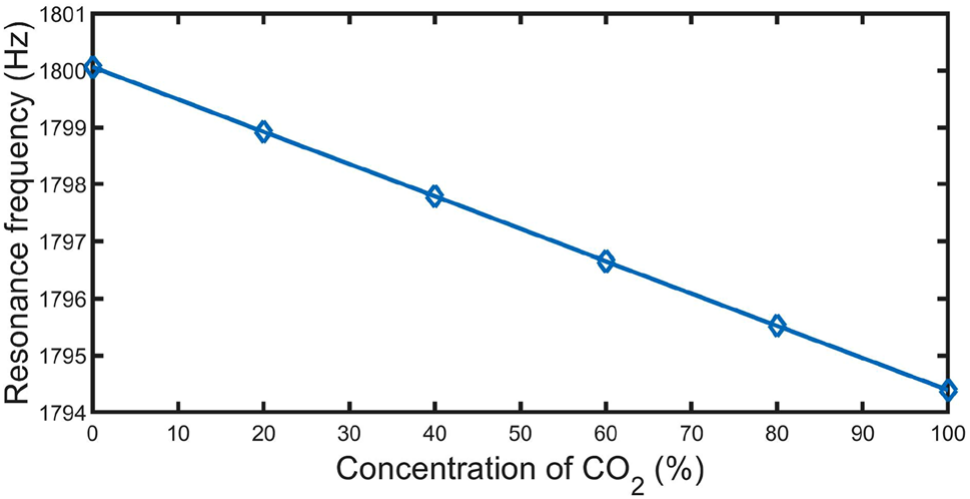
Resonant frequency of localized peak versus ${CO}_{2}$ concentration.

Besides, a linear fitting is performed, as clear in Fig. 12, between the ${CO}_{2}$ concentration ($\alpha$) inside the DEB sample and ${f}_{R}$. According to (27), the resonant frequency decreases linearly with increasing the concentration of ${CO}_{2}$. As a result, the concentration of ${CO}_{2}$ within DEB can easily be calculated by determining the position of the resonant frequency.

29 $${f}_{R}= -0.0567\alpha +1800.$$

Table 2 compares the results using this model (open and closed periodic parallel resonators) and a model using regular tubes with different cross-sections^27^. Using open and closed parallel resonators enhanced the efficiency of the gas detector. Even though the sensitivity of open and closed parallel resonators is close to the sensitivity of closed parallel resonators and branched open resonators separately, the FoM of the current study is seventy-three times higher than the FoM of the branched open resonators. Besides, the FoM of the current study is thirty times higher than the FoM of the closed parallel resonators. Also, the Q-factor of the current study is seven 100-fold higher than the Q-factor of the branched open resonators, and 30-fold higher than the Q-factor of the closed parallel resonators. Besides, the combination of open and closed resonators enhanced the linearity, as clear in Fig. 11B in Ref. ^27^ and Fig. 9B in Ref.^45^.

**Table 2:** Comparison study.

Ref	S Hz.s.m^–1^	Q-factor	FoM s.m^–1^	Structure
2022, Ref.^27^	2.55	4077	9.16	Regular tubes with different cross-sections
2023, Ref.^44^	1.58	6790	33.7	Ternary-symmetric periodic tubes
2023, Ref.^45^	5.8	5000	140	Branched open resonators
2024, Ref.^46^	4.1	113,962	332	Closed parallel resonators
This study	5.3	3,470,288	10,254	Open and closed parallel resonators

The main issue with the proposed structure is that it may be impractical when considering the size of the overall structure. Thus, we will attempt to decrease the model's size in subsequent work.

Finally, it is undeniable that the experimental transmittance may slightly decrease due to viscous losses, thermal losses, or various relaxation effects^47,48^. However, the sensor's sensitivity will not change because this effect will be constant, and this loss will not affect the resonant peak shift. Besides, the step width in almost all figures is high because the resonant peaks appear at specific frequencies, not continuously.

## Conclusion

This study proposed a novel diagnostic biosensor using a periodic band gap design for detecting ${CO}_{2}$ in dry exhaled breath as a biomarker of diseases like diabetes, cancer, and infectious diseases. After optimization of different parameters such as d and S of tubes, the sensitivity, FoM, and Q-factor can reach 5.3 $Hz.{m}^{-1}.s$, 10,254 ${m}^{-1}.s$, and 3,470,288, respectively. Besides, this model achieved high stability in its sensitivity. The suggested sensor has a high sensitivity and simple design, making it suitable for disease detection.

## Data Availability

Requests for materials and data should be addressed to Zaky A. Zaky.
